# Development and validation of a multivariate predictive model for cancer-related fatigue in esophageal carcinoma: a prospective cohort study integrating biomarkers and psychosocial factors

**DOI:** 10.3389/fonc.2025.1674710

**Published:** 2025-10-02

**Authors:** Yunxia Lan, Yongting Wang, Tiantian Jia, Qingyun Cheng, Siyu Han, Yanzhi Mi, Mi Ding

**Affiliations:** ^1^ Department of Nursing, The Affiliated Chest Hospital of Zhengzhou University, Zhengzhou, China; ^2^ Cardiac Surgery Intensive Care Unit, The Affiliated Chest Hospital of Zhengzhou University, Zhengzhou, China; ^3^ Thoracic Surgery Ward 1 and Ward 7, Breast and Thyroid Surgery Department, The Affiliated Chest Hospital of Zhengzhou University, Zhengzhou, China; ^4^ Thoracic Surgery, Henan Cancer Hospital, Zhengzhou, China

**Keywords:** esophageal carcinoma, cancer-related fatigue, predictive model, nomogram, psychosocial factors, biomarkers

## Abstract

**Background:**

To develop and validate a predictive model for cancer-related fatigue (CRF) in patients with esophageal cancer.

**Methods:**

A convenience sample comprising patients diagnosed with esophageal cancer and admitted to the Department of Thoracic Surgery at a tertiary hospital in Henan Province, China, between June 2024 and May 2025, was enrolled. Data were collected using a general information questionnaire, the Chinese version of the revised Piper Fatigue Scale, the Hospital Anxiety and Depression Scale, the Pittsburgh Sleep Quality Index, the Nutrition Risk Screening 2002, and a visual analogue scale. Then, univariate and multivariate logistic regression analyses were conducted to identify risk factors and construct the predictive model. Lastly, a nomogram was developed, and its performance was evaluated through internal and external validation.

**Results:**

The incidence of CRF among patients with esophageal cancer was 70.67%. Multivariate logistic regression identified preoperative hemoglobin concentration, postoperative day-1 serum potassium level, neutrophil ratio, nutritional impairment, anxiety, depression, and sleep disturbance as independent risk factors (all *p* < 0.05). The model demonstrated satisfactory discriminatory power, with a sensitivity of 90.60% and specificity of 93.44%.Additionally, the Hosmer-Lemeshow test indicated favorable calibration (*χ²* = 7.048; *p* = 0.531). In the validation cohort, the area under the receiver operating characteristic curve was 0.887 (95% *CI* 0.802-0.944), with an optimal cut-off value of 0.797, yielding a sensitivity of 82.54% and specificity of 81.48%. Finally, calibration plots revealed excelling agreement between predicted and observed outcomes, and decision curve analysis suggested favorable clinical utility.

**Conclusion:**

The proposed model reliably predicts the risk of cancer-related fatigue in patients with esophageal cancer and may assist in the early identification of high-risk individuals, thereby enabling timely and targeted interventions.

## 1 Introduction

Cancer-related fatigue (CRF) is defined as a subjective sense of physical, cognitive, or emotional tiredness that is related to the disease itself or its treatment, yet disproportionate to recent activity levels (1). Mounting evidence suggests that the prevalence of CRF in patients with esophageal cancer is as high as 88.96%, with severe fatigue being the most prevalent manifestation (2). Compared with other cancer-related symptoms such as pain, depression, or nausea, CRF is more persistent, less responsive to treatment, and exerts a profound negative impact on patients’ quality of life. Previous studies have shown that the occurrence of CRF in patients with esophageal cancer is related to multiple factors, including demographic characteristics (such as gender, age, marital status, economic income, etc.), disease-related factors (such as tumor stage, nutritional status, treatment methods and their side effects) (3, 4). However, there is still a lack of systematic research and clear conclusions on what are the independent risk factors for CRF. Therefore, identifying and verifying its independent risk factors is of great significance for achieving precise intervention.

Currently, clinical assessment of cancer-related fatigue mainly relies on subjective scales. These tools can identify whether a patient is fatigued and the degree of fatigue, but they have limitations in predicting the risk of CRF and screening high-risk populations, and lack comprehensiveness and foresight. In contrast, prediction models constructed based on multivariate analysis can integrate multidimensional information to quantitatively assess a patient’s risk of developing CRF, providing early warning and scientific basis for clinical practice. This study screened and analyzed common factors of CRF in esophageal cancer patients, identified independent risk factors for CRF in esophageal cancer patients, and constructed a risk prediction model, aiming to provide a more scientific, accurate, and convenient new assessment tool for esophageal cancer patients.

## 2 Methods

### 2.1 Study population

This cross-sectional study was conducted in the Department of Thoracic Surgery at a tertiary hospital in Henan Province, China, from June 2024 to May 2025. Eligible participants were required to meet the following inclusion criteria: (1) aged 18 years or older; (2) diagnosed with esophageal cancer based on the diagnostic criteria outlined in the *Chinese Guidelines for Screening and Early Detection of Esophageal Cancer (2022, Beijing)* (5), confirmed by upper gastrointestinal barium swallow, contrast-enhanced thoracic CT, and histopathological biopsy; (3)scheduled to undergo elective combined mediastinoscopic and laparoscopic radical oesophagectomy.

Exclusion criteria were as follows: (1) coexisting coagulation or hematological disorders; (2) history of other surgical interventions within the past 3 months; (3)severe hepatic or renal dysfunction; (4) severe hypertension; (5) diagnosed psychiatric illness or cognitive impairment; (6) concurrent malignancies. A total of 35 variables were included in this study. According to the commonly used empirical rules for logistic regression modeling, the sample size should be 5–10 times the number of candidate variables, while taking into account a 10% sample loss rate, that is: 35×(5~10)/(1-10%)≈194~389 cases. We ultimately included 300 patients, which can meet the sample size requirements for the establishment of the prediction model. The incidence of CRF is 70.67%. The final multivariate model contains only 7 independent predictors. Participants were randomly assigned to a model training group (n=210, 70%) and a validation group (n=90, 30%).

### 2.2 Definition and classification of cancer-related fatigue

CRF was defined in accordance with the 2018 National Comprehensive Cancer Network (NCCN) guidelines (6) as a distressing, persistent, and subjective sense of physical, emotional, and/or cognitive fatigue related to cancer or its treatment, disproportionate to recent activity and interfering with usual functioning. Patients were categorized into a fatigue group or a non-fatigue group based on the presence or absence of CRF.

### 2.3 Assessment instruments

#### 2.3.1 General and clinical data questionnaire

General demographic information include gender, age, marital status, number of children, education level, occupation, medical insurance type, and monthly income. Disease-related information include esophageal cancer stage, tumor progression or recurrence, concomitant diseases, adverse reactions, preoperative hemoglobin concentration, hemoglobin concentration one day after surgery, preoperative albumin level, albumin level one day after surgery, preoperative potassium level, potassium level one day after surgery, preoperative calcium level, calcium level one day after surgery, preoperative sodium level, sodium level one day after surgery, preoperative white blood cell count, white blood cell count one day after surgery, preoperative neutrophil/lymphocytes ratio, and neutrophil/lymphocytes ratio one day after surgery.

#### 2.3.2 Revised Piper Fatigue Scale - Chinese version

The Revised Piper Fatigue Scale-Chinese version (RPFS-CV), translated in 2023 by Hong Kong scholars (7), consists of 27 items (22 scored items and 5 open-ended questions). Item 1 assesses the presence of fatigue; if fatigue is reported, the remaining items are completed by the respondent. Items 3–24 are categorized into four dimensions: behavioral/severity (Items3-8), affective (9-13), sensory (14-18), and cognitive/mood (19-24). Each item is scored on a scale from 0 to 10, with higher scores indicating greater fatigue severity. The average score of all items represents overall fatigue severity. Fatigue severity is categorized as: none (0), mild (1-3), moderate (4-6), or severe (7-10). The scale has a high internal consistency, with a Cronbach’s *α* > 0·90. This study investigated and evaluated CRF in patients with esophageal cancer on 3^th^ after surgery. In this study, patients with an RPFS-CV average score ≥ 1 were classified as the fatigue group, while patients with an average score equal to 0 were classified as the non-fatigue group. This operational definition has been widely used in previous studies on RPFS-CV and provides a standardized basis for group classification (8).

#### 2.3.3 Hospital Anxiety and Depression Scale

Hospital Anxiety and Depression Scale (HADS) is widely applied to assess anxiety and depression in hospitalized patients. It comprises 14 items, with 7 items each for the anxiety (A) and depression (D) subscales. Scores of 0–7 indicate no symptoms, 8–10 suggest borderline symptoms, and 11–21 indicate definite symptoms. A score of ≥8 on either subscale was considered indicative of anxiety or depression in this study (9).

#### 2.3.4 Pittsburgh Sleep Quality Index

Pittsburgh Sleep Quality Index (PSQI) assesses sleep quality over the past month. It is composed of 19 self-rated items grouped into seven components: subjective sleep quality, sleep latency, sleep duration, habitual sleep efficiency, sleep disturbances, use of sleeping medications, and daytime dysfunction. The global PSQI score ranges from 0 to 21, with higher scores reflecting poorer sleep. A cut-off score >7 was used to indicate sleep disturbance in Chinese populations, with sensitivity of 98.3% and specificity of 90·2% (Kappa = 0·89, *p* < 0.01) (10).

#### 2.3.5 Nutritional Risk Screening 2002

The Nutritional Risk Screening 2002 (NRS-2002), widely recommended for nutritional risk screening in adult inpatients, including those with cancer, comprises three components, namely disease severity, nutritional status, and age. Scores range from 0 to 3 for each domain. A total score ≥3 indicates nutritional risk. Its internal consistency (Cronbach’s *α* = 0.67) and content validity have been validated in previous studies (11).

#### 2.3.6 Visual Analog Scale

The Visual Analog Scale (VAS) is a 10-cm horizontal line employed to assess pain intensity, with “no pain” at the left end and “worst imaginable pain” at the right. Patients are instructed to mark a point on the line that best represents their pain level. Scores are recorded as 0-10, with higher values indicating greater pain. VAS is widely used owing to its simplicity and strong validity and reliability in pain assessment (12).

### 2.4 Statistical analysis

Data entry and management were performed using Epidata 3.1, while statistical analyses for descriptive and group comparisons were conducted using SPSS version 22.0. The *rms* package in R version 4.3.1 was utilized to construct the nomogram.

Continuous variables with a normal distribution were expressed as mean ± SD, while non-normally distributed data were reported as median and interquartile range. Categorical and ordinal variables were described using frequencies and proportions. For comparisons between the training model and validation groups, independent *t* tests were used for normally distributed data, and rank-sum tests were applied for non-normally distributed or ordinal data. Chi-squared, continuity-corrected chi-squared, or Fisher’s exact tests were applied for the comparison of categorical variables. Univariate and multivariate logistic regression analyses were carried out to identify independent predictors of CRF and develop the risk prediction model.

A two-tailed (*p* < 0.05) was considered statistically significant.

### 2.5 Ethical principles and moral declarations norms

This research strictly adhered to the relevant provisions stipulated in the Helsinki Declaration of the World Medical Association. This study was approved by the institutional ethics committee (Approval No. 2024-KL-05-12), and all participants provided written informed consent. The following rules were followed: (1) Principle of informed consent: Before filling out the questionnaire, the background, purpose and content of the study were explained to the patients and their consent was obtained; (2) Principle of respect for human rights: The patients were informed that they could request to suspend or withdraw from the study at any time if they felt any physiological or pathological discomfort or other reasons during the investigation; (3) Principle of confidentiality: The basic information, disease data, examination results and scale assessment results of the patients obtained were only used for this study and the researchers were responsible for keeping the relevant information properly and not disclosing it to others.

## 3 Results

### 3.1 Baseline characteristics of patients with esophageal cancer

A total of 300 eligible patients with esophageal cancer were enrolled in the study, including 210 in the training model group and 90 in the validation group. Demographic characteristics, disease-related variables, and the prevalence of anxiety, depression, sleep disturbance, and nutritional risk were similar between the two groups (all *P* > 0.05), indicating good homogeneity and comparability between groups (**Table 1**).

**Table 1:** Comparison of baseline characteristics between in the training model group and the validation group.

Factor	Categories	Training model group (n=210)	Validation group (n=90)	*t/Z/*c2	*P*
Gender				3.273 ^c^	0.070
	Male	159(75.71)	59(65.56)		
	Female	51(24.29)	31(34.44)		
Age				1.006 ^c^	0.316
	≤60	47(22.38)	25(27.78)		
	>60	163(77.62)	65(72.22)		
Marital Status				3.915 ^c^	0.141
	Married	201(95.71)	82(91.11)		
	Unmarried	0(0)	1(1.11)		
	Lose a Spouse	9(4.29)	7(7.78)		
Number of Children (individuals)				1.600 ^c^	0.659
	0	2(0.95)	0(0)		
	1	19(9.05)	6(6.67)		
	≥2	93(90)	84(93.33)		
Educational Background				3.489 ^c^	0.175
	Middle School and Below	171(81.43)	66(73.33)		
	High School/Vocational School	33(15.71)	18(20)		
	College Degree or Above	6(2.86)	6(6.67)		
Career				1.656 ^c^	0.647
	Farmer	162(77.14)	68(75.56)		
	Workers	21(10)	13(14.44)		
	Retiree	25(11.9)	8(8.89)		
	Other	2(0.95)	1(1.11)		
Medical Insurance Type				1.810 ^c^	0.405
	Urban and Rural Residents’ Medical Insurance	159(75.71)	72(80)		
	Employee Medical Insurance	49(23.33)	16(17.78)		
	Other	2(0.95)	2(2.22)		
Monthly Household Income (yuan)				2.263 ^c^	0.322
	≤.322	26(12.38)	9(10)		
	3000-8000	150(71.43)	60(66.67)		
	>8000	34(16.19)	21(23.33)		
Staging of Esophageal Cancer				0.799 ^c^	0.850
	Phase I	30(14.29)	11(12.22)		
	Phase II	107(50.95)	43(47.78)		
	Phase III	71(33.81)	35(38.89)		
	Phase IV	2(0.95)	1(1.11)		
Tumor progression or recurrence		20(9.52)	11(12.22)	0.495 ^c^	0.482
Merge other diseases		146(69.52)	59(65.56)	0.458 ^c^	0.498
Adverse Reactions Occur		116(55.24)	42(46.67)	1.857 ^c^	0.173
Nutritional Disorders		88(41.9)	39(43.33)	0.053 ^c^	0.818
Anxiety				4.879 ^c^	0.087
	Asymptomatic	59(28.1)	32(35.56)		
	Possible Anxiety	89(42.38)	26(28.89)		
	Anxiety	62(29.52)	32(35.56)		
Depressed				1.178 ^c^	0.555
	Asymptomatic	36(17.14)	12(13.33)		
	Possible Depressed	115(54.76)	48(53.33)		
	Depressed	59(28.1)	30(33.33)		
Sleep Disturbance		139(66.19)	52(57.78)	1.927 ^c^	0.165
Preoperative Hemoglobin Concentration		115.93 ± 18.53	113.21 ± 18.38	1.169 ^a^	0.243
Hemoglobin Concentration One Day after Surgery		118.51 ± 14.51	118.93 ± 15.95	0.222 ^a^	0.824
Preoperative Albumin Level		366.65 ± 87.76	347.56 ± 109.39	1.600 ^a^	0.111
Albumin Level 1 Day after Surgery		345.56 ± 43.62	342.74 ± 47.26	0.499 ^a^	0.618
Preoperative Potassium Ion Level		407.07 ± 39.62	413.93 ± 44.61	1.323 ^a^	0.187
Potassium Ion Level 1 Day after Surgery		419.13 ± 55.47	409.07 ± 42.11	1.541 ^a^	0.124
Preoperative Calcium Ion Level		207.79 ± 59.87	213.11 ± 52.34	0.732 ^a^	0.465
Calcium Ion Level 1 Day after Surgery		203.52 ± 40.48	204.32 ± 41.16	0.157 ^a^	0.876
Preoperative Sodium Ion Level		140.00 ± 3.12	140.26 ± 3.11	0.651 ^a^	0.515
Sodium Ion Level 1 Day after Surgery		137.84 ± 3.16	137.86 ± 3.05	0.044 ^a^	0.965
Preoperative White Blood Cell Count		508(388,668)^d^	545(417,668)^d^	0.429 ^b^	0.668
White Blood Cell Count One Day after Surgery		1177(970,1380)^d^	1124(918,1417)^d^	0.807 ^b^	0.420
Preoperative Neutrophil Ratio (x103)		0.62(0.55,0.71)	0.62(0.57,0.78)^d^	0.592 ^b^	0.554
Neutrophil Ratio on Postoperative Day 1(x10^3^)		0.92(0.88,8.27)^d^	1.61(0.88,8.88)^d^	1.468 ^b^	0.142
VAS Scores		3.19 ± 1.16	3.04 ± 1.08	0.988 ^a^	0.324

^a^ *t* test; ^b^ *Mann-Whitney U rank-sum* test; ^c^ *χ²* test; ^d^(*M* (*P*25,*P*75).

### 3.2 Incidence of cancer-related fatigue in patients with esophageal cancer

Based on the presence or absence of cancer-related fatigue (CRF), patients were assigned to a fatigue group and a non-fatigue group. Among the 300 patients, 212 (70.67%) experienced CRF. Specifically, 149 patients (70.95%) and 63 patients (70.00%) experienced CRF in the training model and validation groups, respectively.

### 3.3 Univariate analysis of factors associated with cancer-related fatigue

Univariate analysis revealed that monthly household income, preoperative hemoglobin concentration, postoperative day-1 serum potassium level, neutrophil ratio, nutritional risk, anxiety, depression, and sleep disturbance were significantly associated with the development of CRF (*p* < 0.05; **Table 2**).

**Table 2:** Univariate analysis of factors associated with cancer-related fatigue.

Factor	Categories	Non-fatigue group (n=61)	Fatigue group (n=149)	*t/Z/*c2	*P*
Gender				1.275 ^c^	0.259
	Male	43(70.49)	116(77.85)		
	Female	18(29.51)	33(22.15)		
Age				2.514 ^c^	0.113
	≤60	18(29.51)	29(19.46)		
	>60	43(70.49)	120(80.54)		
Marital Status				3.206 ^c^	0.073
	Married	56(91.8)	145(97.32)		
	Unmarried	5(8.2)	4(2.68)		
	Lose a Spouse	0(0)	2(1.34)		
Number of Children (individuals)				2.217 ^c^	0.529
	0	5(8.2)	14(9.4)		
	1	24(39.34)	69(46.31)		
	≥2	32(52.46)	64(42.95)		
Educational Background					
	Middle School and Below			1.744 ^c^	0.418
	High School/Vocational School	53(86.89)	118(79.19)		
	College Degree or Above	7(11.48)	26(17.45)		
Career		1(1.64)	5(3.36)		
	Farmer			1.522 ^c^	0.677
	Workers	50(81.97)	116(77.85)		
	Retiree	4(6.56)	15(10.07)		
	Other	7(11.48)	16(10.74)		
Medical Insurance Type		0(0)	2(1.34)		
	Urban and Rural Residents’ Medical Insurance			3.301 ^c^	0.192
	Employee Medical Insurance	51(83.61)	108(72.48)		
	Other	10(16.39)	39(26.17)		
Monthly Household Income (yuan)		0(0)	2(1.34)		
	≤(1.3			15.343 ^c^	<0.001
	3000-8000	0(0)	26(17.45)		
	>8000	54(88.52)	96(64.43)		
Staging of Esophageal Cancer		7(11.48)	27(18.12)		
	Phase I			4.259 ^c^	0.235
	Phase II	8(13.11)	22(14.77)		
	Phase III	37(60.66)	70(46.98)		
	Phase IV	15(24.59)	56(37.58)		
Tumor progression or recurrence		1(1.64)	1(0.67)		
Merge other diseases		8(13.11)	12(8.05)	1.287 ^c^	0.257
Adverse Reactions Occur		45(73.77)	101(67.79)	0.732 ^c^	0.392
Nutritional Disorders		39(63.93)	77(51.68)	2.63 ^c^	0.105
Anxiety		14(22.95)	74(49.66)	12.687 ^c^	<0.001
	Asymptomatic			61.52 ^c^	<0.001
	Possible Anxiety	40(65.57)	19(12.75)		
	Anxiety	16(26.23)	73(48.99)		
Depressed		5(8.2)	57(38.26)		
	Asymptomatic			35.166 ^c^	<0.001
	Possible Depressed	24(39.34)	12(8.05)		
	Depressed	31(50.82)	84(56.38)		
Sleep Disturbance		6(9.84)	53(35.57)		
Preoperative Hemoglobin Concentration		21(34.43)	118(79.19)	38.762 ^c^	<0.001
Hemoglobin Concentration One Day after Surgery		127.43 ± 15.59	111.23 ± 17.60	6.251 ^a^	<0.001
Preoperative Albumin Level		118.30 ± 14.03	118.60 ± 14.75	0.140 ^a^	0.889
Albumin Level 1 Day after Surgery		374.34 ± 72.00	363.50 ± 93.49	0.812 ^a^	0.418
Preoperative Potassium Ion Level		347.95 ± 45.24	344.58 ± 43.06	0.508 ^a^	0.612
Potassium Ion Level 1 Day after Surgery		409.23 ± 40.62	406.19 ± 39.31	0.504 ^a^	0.615
Preoperative Calcium Ion Level		445.38 ± 65.84	408.38 ± 46.76	4.594 ^a^	<0.001
Calcium Ion Level 1 Day after Surgery		201.36 ± 65.43	210.42 ± 57.46	0.995 ^a^	0.321
Preoperative Sodium Ion Level		200.77 ± 44.08	204.64 ± 39.01	0.629 ^a^	0.530
Sodium Ion Level 1 Day after Surgery		139.84 ± 2.97	140.07 ± 3.18	0.487 ^a^	0.627
Preoperative White Blood Cell Count		138.08 ± 3.25	137.74 ± 3.13	0.715 ^a^	0.476
White Blood Cell Count One Day after Surgery		506(388,640)^d^	509(392,683)^d^	0.294 ^b^	0.769
Preoperative Neutrophil Ratio (x103)		1186(999,1428)^d^	1174(953,1376)^d^	0.846 ^b^	0.398
Neutrophil Ratio on Postoperative Day 1(x10^3^)		0.61(0.52,0.71)^d^	0.63(0.55,0.71)^d^	1.184 ^b^	0.236
VAS Scores		0.91(0.87,2.80)^d^	0.92(0.88,8.80)^d^	2.569 ^b^	0.010

^a^ *t* test; ^b^ *Mann-Whitney U rank-sum* test; ^c^ *χ²* test; ^d^(*M* (*P*25,*P*75).

### 3.4 Multivariate analysis of factors associated with cancer-related fatigue in patients with esophageal cancer

Next, the eight variables identified as significant in univariate analysis (*p* < 0.05) were incorporated as independent variables in a multivariate logistic regression model, with the presence of cancer-related fatigue as the dependent variable. The coding scheme of independent variables is listed in **Table 3**.

**Table 3:** Variable coding scheme.

Risk factors	Variable	Assignment
Tired	Y	No=0, Yes=1
Monthly Household Income (yuan)	X_1_	3000 below=1,3000 to 8000 = 2,8000 above=3
Preoperative Hemoglobin Concentration	X_2_	actual value
Potassium Ion Level 1 Day after Surgery	X_3_	actual value
Neutrophil Ratio on Postoperative Day 1 (×10³)	X_4_	actual value
Nutritional Disorders (score)	X_5_	<3 = 0 (no nutritional risk), ≥ 3 = 1 (nutritional risk present)
Anxiety (score)	X_6_	<7 = 1 (no anxiety symptoms), 8-10 = 2 (possible anxiety), ≥ 11 = 3 (anxiety)
Depression (score)	X_7_	<7 = 1 (no depressive symptoms), 8-10 = 2 (possible depression), ≥ 11 = 3 (depression)
Sleep Disorders	X_8_	≤ 7 = 0 (no sleep disorders),>7 = 1 (presence of sleep disorders)

As anticipated, the results indicated that preoperative hemoglobin concentration, postoperative day-1 serum potassium level, neutrophil ratio, nutritional risk, anxiety, depression, and sleep disturbance were independent risk factors for cancer-related fatigue in patients with esophageal cancer (*p* < 0.05) (**Table 4**).

**Table 4:** Results of multivariate binary logistic regression analysis.

Risk Factors	B	S.E	Wald	*P*	*OR*	*95%CI*
Monthly Household Income (yuan)	-0.391	0.508	0.592	0.442	0.676	0.25-1.832
Preoperative Hemoglobin Concentration	-0.070	0.019	13.507	<0.001	0.932	0.898-0.968
Potassium Ion Level 1 Day after Surgery	-0.013	0.006	5.505	0.019	0.987	0.977-0.998
Neutrophil Ratio on Postoperative Day 1 (×10³)	0.328	0.124	7.002	0.008	1.388	1.089-1.769
Nutritional Disorders (score)	1.215	0.584	4.334	0.037	3.370	1.074-10.58
Anxiety (score)	1.940	0.431	20.282	<0.001	6.956	2.991-16.181
Depression (score)	1.879	0.539	12.141	<0.001	6.547	2.275-18.841
Sleep Disorders	2.176	0.573	14.437	<0.001	8.810	2.868-27.069
Constant	5.632	3.602	2.445	0.118		

### 3.5 Nomogram for predicting the risk of cancer-related fatigue in patients with esophageal cancer

Furthermore, a risk prediction model was constructed using the independent risk factors identified for cancer-related fatigue in patients with esophageal cancer. The predictive formula was as follows: Logit *P* = 4.843−0.07×preoperative hemoglobin concentration−0.113×serum potassium level on postoperative day 1 + 0.328×neutrophil ratio on postoperative day 1(×10³)+1.168 nutritional risk+1.919×anxiety+ 1.919×depression+2.107×sleep disturbance.

**Figure 1:**
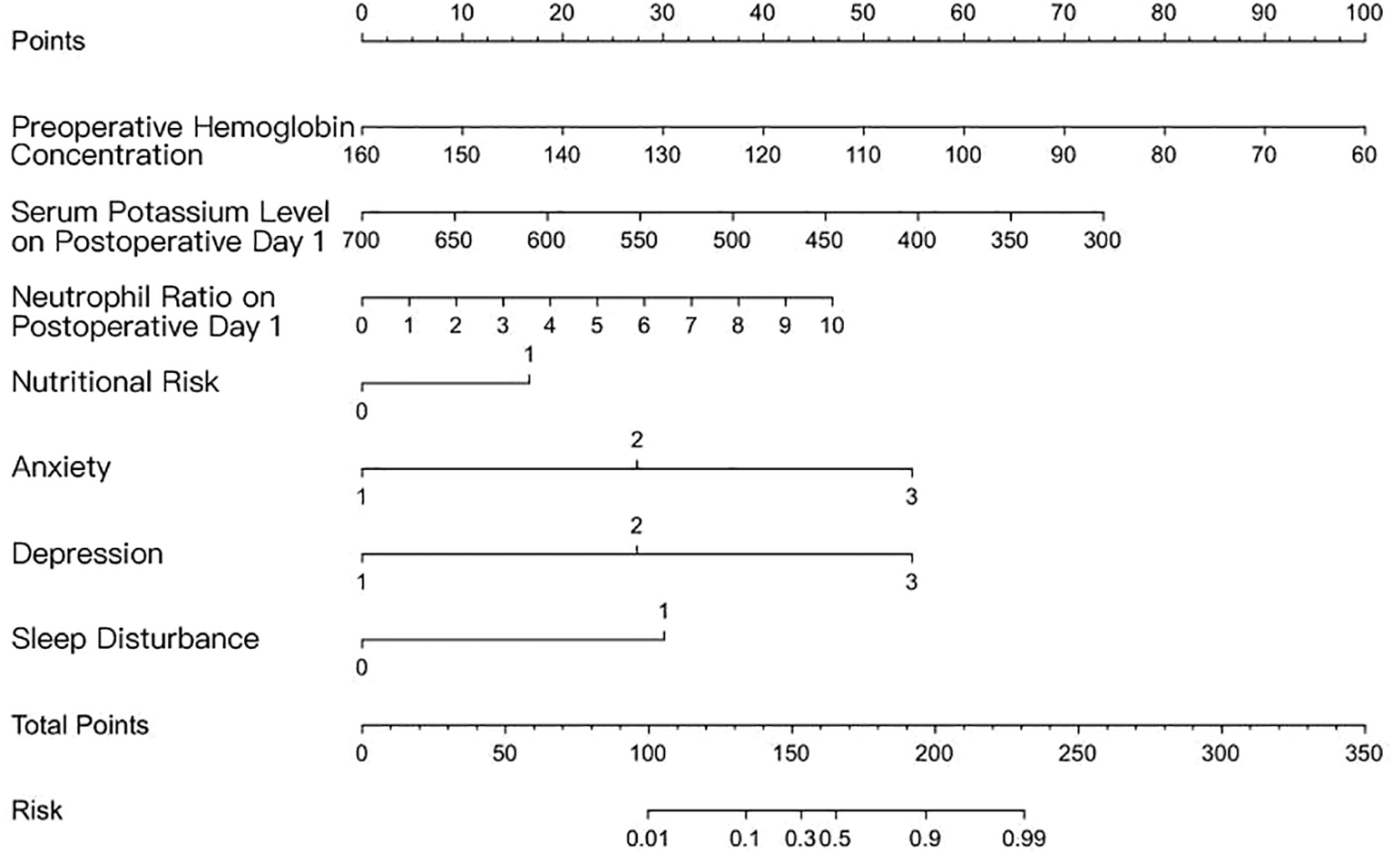
Nomogram for predicting the risk of cancer-related fatigue in patients with esophageal cancer.

After stepwise forward selection, this model remained unchanged. To facilitate clinical interpretation, we provide a sample for the risk estimate. Assume a patient has the following characteristics: preoperative hemoglobin: 130 g/L corresponds to 30 points; serum potassium on postoperative day 1: 500 corresponds to 40 points; neutrophil count on postoperative day 1: 4 corresponds to 20 points; nutritional risk: 0 corresponds to 0 points; anxiety: 2 corresponds to 50 points; depression: 1 corresponds to 30 points; and sleep disturbance: 0 corresponds to 0 points. To calculate total points, add the points for each variable: 30 + 40 + 20 + 0 + 50 + 30 + 0 = 170 total points. Find 170 on the total points axis, map it vertically to the risk axis, and read the risk probability: 170 total points corresponds to a risk of approximately 30%. (Since a total point of 150 corresponds to 0.3, 170 is close to this value, so the risk is approximately 30%.). The model was visualized as a nomogram, as presented in **Figure 1**.

### 3.6 Internal validation of the model

The model was internally validated using the bootstrap method. The concordance index (C-index) was 0.962, and the area under the receiver operating characteristic curve (AUC) was 0.962 (95% CI 0.926-0.983), indicating an outstanding level of discrimination between patients with and without fatigue. At a cut-off value of 0.715, the Youden index was maximized at 0.840, corresponding to a sensitivity of 90.60% and specificity of 93.44% (**Table 5**).

**Table 5:** ROC curve analysis of the cancer-related fatigue prediction model in patients with esophageal cancer.

AUC	SE	95%CI	*Z*	*P*	Relevant standards	Sensitivity (%)	Specificity (%)	Youden index J
0.962	0.014	0.926 - 0.983	33.686	<0.001	>0.715	90.60	93.44	0.841

**Figure 2:**
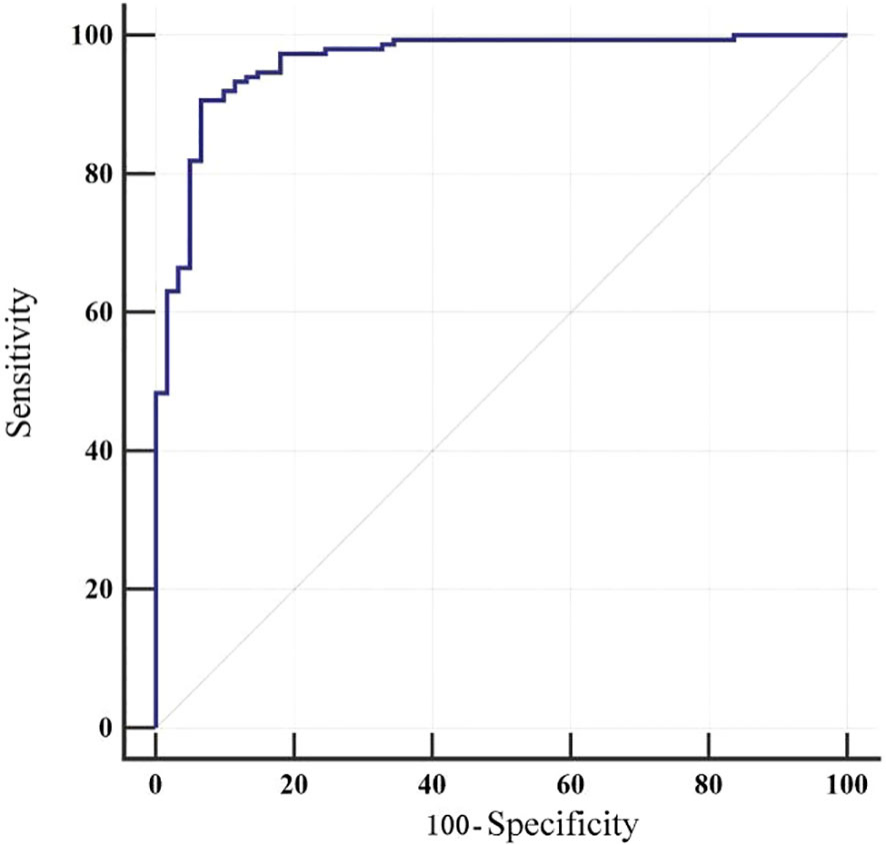
ROC curve for internal evaluation of the model.

The Hosmer-Lemeshow goodness-of-fit test yielded *χ²* = 7.048 (*p* = 0.531), indicating good model calibration. The receiver operating characteristic (ROC) curve is illustrated in **Figure 2**.

### 3.7 External validation of the model

**Figure 3:**
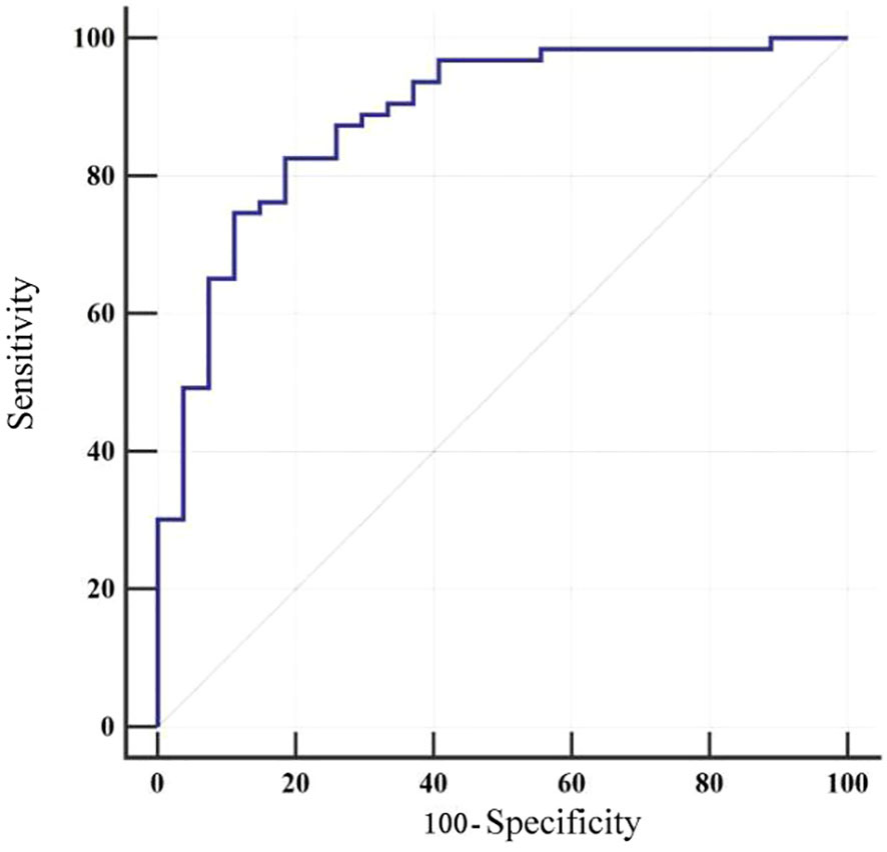
ROC curve for external validation of the model.

The model was applied to calculate the predicted probability of fatigue for each patient in the validation cohort. Using the actual fatigue status of these patients, a receiver operating characteristic (ROC) curve was plotted (**Figure 3**) to assess the discriminative ability of the model. The area under the ROC curve (AUC) was 0.887 (95%*CI* 0.802-0.944). Meanwhile, at the optimal cut-off value of 0.797, the model achieved a sensitivity of 82.54% and specificity of 81.48%, highlighting the clinical utility of the model (**Table 6**).

**Table 6:** ROC curve analysis for external validation of the cancer-related fatigue prediction model in patients with esophageal cancer.

AUC	SE	95%CI	*Z*	*P*	Relevant standards	Sensitivity (%)	Specificity (%)	Youden index J
0.887	0.038	0.802 - 0.944	10.054	<0.001	>0.797	82.54	81.48	0.640

## 4 Discussion

### 4.1 High incidence of cancer-related fatigue in patients with esophageal cancer

The reported incidence of cancer-related fatigue (CRF) among patients with esophageal cancer varies across studies both domestically and internationally. Herein, its incidence was 70.67%. At the same time, Li Yamin documented an incidence of 88.96% in Hebei Province, predominantly severe fatigue ^[2]^. Likewise, Li Wen reported a postoperative 1–3 day CRF incidence as high as 89.6% (13). Indeed, CRF is highly prevalent in this patient population.

As a subjective experience, CRF relies on patient self-report. Nonetheless, patients generally lack awareness of CRF-related symptoms and objective standards to quantify their fatigue. Notably, fatigue is frequently considered a normal, inevitable symptom during treatment and thus seldom reported to clinicians, reflecting a general deficiency in patient knowledge. Consequently, healthcare professionals may underestimate CRF, leading to clinical oversight. Therefore, it is crucial to recognize and identify potential risk factors for CRF in patients with esophageal cancer. Enhancing awareness and providing proactive care for high-risk patients is vital for improving patient prognosis and overall quality of life.

### 4.2 Interaction of risk factors in the predictive model and implications for preventive strategies

Preoperative hemoglobin concentration, indicated by low hemoglobin concentration, was identified as a direct risk factor for CRF. A retrospective cohort study recruiting 647 patients undergoing radical surgery for esophageal cancer reported a 33.6% prevalence of preoperative hemoglobin concentration (14). Herein, preoperative hemoglobin levels were higher in the non-fatigue group compared to the fatigue group. Rapid tumor growth and extensive use of antimetabolic agents during cancer treatment deplete folate and impair bone marrow hematopoiesis, thereby reducing peripheral hemoglobin and erythrocyte counts. Of note, perioperative transfusion rates, postoperative complications, and hospital stays are more likely to be higher in patients with preoperative hemoglobin concentration. Earlier meta-analyses have described that recombinant human erythropoietin effectively elevates preoperative hemoglobin levels and concurrently minimizes the need for perioperative transfusion (15, 16). Thus, close monitoring and timely, precise clinical interventions for patients with low preoperative hemoglobin are warranted to improve postoperative outcomes.

At present, surgery and chemotherapy remain the primary treatment modalities for esophageal cancer. Poor nutritional status compromises wound healing and granulation tissue formation, while postoperative gastrointestinal decompression can result in substantial fluid loss, leading to hypokalemia and increasing the risk of CRF.

The interplay between inflammation and malignancy is well recognized (17). Systemic inflammatory responses not only promote or inhibit tumor development but also affect patient responses to systemic anti-tumor therapies. Neutrophil counts, a common clinical inflammatory marker, reflect the internal homeostasis and tumor progression. More importantly, neutropenia predisposes patients to infection and inflammation, while chronic inflammation can induce CRF (18, 19). Studies have shown that nasopharyngeal carcinoma patients have CRF after chemotherapy, and CRF gradually worsens during concurrent chemoradiotherapy. Neutrophil/lymphocyte ratio and high-sensitivity C-reactive protein level will also gradually increase. Neutrophil/lymphocyte ratio and high-sensitivity C-reactive protein level are significantly positively correlated with CRF score (20). Similarly, the results of this study showed a significant positive correlation between the neutrophil/lymphocyte ratio and the development of CRF one day after surgery. This suggests that both decreased immune function due to low neutrophil counts and inflammatory activation reflected by elevated neutrophil ratios may contribute to the development and progression of CRF through different pathways. Abnormal neutrophil levels themselves are a potential risk factor for CRF, suggesting that healthcare professionals should dynamically monitor inflammatory markers in clinical practice, identify high-risk patients early, and intervene to improve their quality of life and prognosis.

### 4.3 Predictive performance of the cancer-related fatigue risk model

Previous CRF models for esophageal cancer have employed a retrospective design, which is subject to recall bias and missing variables (e.g., some psychological indicators are not recorded). Most models focus on “clinical pathology indicators and a single biomarker,” neglecting psychological-physiological interactions. This study employed a prospective, dynamic model to avoid retrospective bias, enabling real-time updates of risks during treatment and promoting a transition from static assessment to dynamic monitoring. This study developed and externally validated a risk prediction model for CRF in patients with esophageal cancer. Noteworthily, the area under the curve (AUC) exceeded 0.8 in both the training and validation cohorts. Besides, the calibration curves were closely aligned with the ideal reference line, and Hosmer-Lemeshow tests yielded *p* > 0.05, demonstrating good discrimination, calibration, and clinical utility. Visualization of the model as a nomogram facilitates intuitive interpretation and calculation, thereby enhancing clinical applicability. The model’s simple, easily understandable items enable administration through verbal questioning, suitable for elderly patients and ensuring high accessibility. However, this study still has certain limitations, and subsequent research needs to address both spatial representativeness and temporal continuity. First, there are limitations to model validation. The validation and modeling cohorts were both from the same medical institution. Due to the high homogeneity of disease characteristics, treatment processes, and medical resource allocation among patients within a single institution, the model’s applicability to other medical centers or the broader esophageal cancer population may be limited. The validation approach, which employed internal data splitting, does not constitute “true external validation” across multiple centers or diverse clinical settings. Its generalizability requires the inclusion of more heterogeneous study populations and treatment settings. Second, there are limitations to the study design and follow-up, including the lack of long-term dynamic follow-up of CRF. CRF is a dynamic symptom that is closely related to factors such as treatment progress and disease progression. Prediction models based solely on single or short-term assessments are unlikely to reflect the dynamic evolution of fatigue. The long-term predictive power for fatigue requires further exploration, including repeated assessments of CRF at key postoperative time points (e.g., 1 week, 2 weeks, and 1 month) to construct dynamic prediction models to enhance the clinical utility of the study’s findings.

## 5 Conclusion

In this study, the prevalence of cancer-related fatigue (CRF) among patients with esophageal cancer was found to be 70.67%, underscoring its high burden in this population. We identified several independent risk factors for CRF, including preoperative hemoglobin concentration, postoperative day-1 serum potassium levels, neutrophil ratio, nutritional impairment, anxiety, depression, and sleep disturbance. Based on these variables, we developed and validated a risk prediction model that demonstrated good accuracy, discrimination, and clinical utility.

This model provides a practical tool for the early identification of patients at high risk of CRF, enabling timely and individualized interventions. Moreover, the recognition of specific independent risk factors offers valuable insights for tailoring clinical management strategies aimed at improving quality of life in esophageal cancer patients.

The study therefore confirms its initial hypothesis and successfully achieves its objective of clarifying the independent risk factors for CRF and establishing a predictive model. Nevertheless, limitations regarding the scope of predictor variables and the representativeness of the study cohort should be acknowledged. Future studies with larger and more diverse populations, as well as comprehensive variable collection, are warranted to further refine and validate the model.

## Data Availability

The raw data supporting the conclusions of this article will be made available by the authors, without undue reservation.
